# COVID-19 and Breast Cancer: Analysis of Surgical Management of a Large Referral Center during the 2020–2021 Pandemic Period

**DOI:** 10.3390/curroncol30050359

**Published:** 2023-05-05

**Authors:** Fulvio Borella, Luca Bertero, Fabrizia Di Giovanni, Gianluca Witel, Giulia Orlando, Alessia Andrea Ricci, Alessandra Pittaro, Isabella Castellano, Paola Cassoni

**Affiliations:** 1 Obstetrics and Gynecology Unit 1, Department of Surgical Sciences, University of Turin, 10126 Turin, Italy; fulvio.borella87@gmail.com; 2 Pathology Unit, Department of Medical Sciences, University of Turin, 10126 Turin, Italy; 3 Pathology Unit, Department of Oncology, University of Turin, 10126 Turin, Italy; 4 Pathology Unit, Città della Salute e della Scienza University Hospital, 10126 Turin, Italy

**Keywords:** COVID-19, SARS-CoV-2, breast cancer, surgery, pandemic, management, screening

## Abstract

Background: Coronavirus disease-19 (COVID-19) has spread worldwide since December 2019 and was officially declared a pandemic in March 2020. Due to the rapid transmission and the high fatality rate, drastic emergency restrictions were issued, with a negative impact on routine clinical activities. In particular, in Italy, many authors have reported a reduction in the number of breast cancer diagnoses and critical problems in the management of patients who accessed the breast units during the dramatic first months of the pandemic. Our study aims to analyze the global impact of COVID-19 in the two years of the pandemic (2020–2021) on the surgical management of breast cancer by comparing them with the previous two years. Methods: In our retrospective study, we analyzed all cases of breast cancer diagnosed and surgically treated at the breast unit of “Città della Salute e della Scienza” in Turin, Italy, making a comparison between the 2018–2019 pre-pandemic period and the 2020–2021 pandemic period. Results: We included in our analysis 1331 breast cancer cases surgically treated from January 2018 to December 2021. A total of 726 patients were treated in the pre-pandemic years and 605 in the pandemic period (−121 cases, 9%). No significant differences were observed regarding diagnosis (screening vs. no screening) and timing between radiological diagnosis and surgery for both in situ and invasive tumors. There were no variations in the breast surgical approach (mastectomy vs. conservative surgery), while a reduction in axillary dissection compared to the sentinel lymph node in the pandemic period was observed (*p*-value < 0.001). Regarding the biological characteristics of breast cancers, we observed a greater number of grades 2–3 (*p*-value = 0.007), pT stage 3–4 breast cancer surgically treated without previous neoadjuvant chemotherapy (*p*-value = 0.03), and a reduction in luminal B tumors (*p*-value = 0.007). Conclusions: Overall, we report a limited reduction in surgical activity for breast cancer treatment considering the entire pandemic period (2020–2021). These results suggest a prompt resumption of surgical activity similar to the pre-pandemic period.

## 1. Introduction

Coronavirus disease-19 (COVID-19), the infectious disease caused by the novel severe acute respiratory syndrome coronavirus 2 (SARS-CoV-2), was first reported in Wuhan (China) in December 2019 and officially declared as a pandemic by the World Health Organization (WHO) on 11 March 2020 [1].

The transmission of SARS-CoV-2 mainly occurs through respiratory droplets and aerosols that are generated when an infected person coughs, sneezes, or talks. The virus can also spread through contact with contaminated surfaces or objects. The incubation period of COVID-19 ranges from 1 to 14 days, with a median of 5 days. The symptoms of COVID-19 vary from mild to severe and include fever, cough, shortness of breath, loss of taste or smell, headache, fatigue, and diarrhea. Some people may develop more serious complications, such as pneumonia, acute respiratory distress syndrome, septic shock, multiorgan failure, and death [2].

In March 2020, due to the rapid spread of SARS-CoV-2 and the high mortality especially observed among frail and elderly patients, drastic and urgent measures were introduced in Italy. These severe measures to contain the spread of SARS-CoV-2 have led to the cessation of most work activities, local school closures, quarantine for those affected by COVID-19, and social isolation [3,4]. The Italian health system, which offers universal access to health care, faced enormous pressure from the pandemic. The National Healthcare Service was close to collapse in the most affected regions, especially Lombardy, where hospitals were overwhelmed by the number and severity of cases [5]. The health system faced a shortage of medical supplies, equipment, and personnel, as well as a surge in hospitalizations and intensive care admissions [5]. Health workers were exposed to a high risk of infection and burnout while trying to provide care under challenging conditions [6,7]. In this context, several hospitals had been converted into centers for the treatment of COVID-19 with the relocation of specialist personnel and a slowdown in other medical activities. In particular, the management of patients affected by breast cancer (BC) suffered the effect of the pandemic, with a reduction in BC screening, diagnoses, and consequently access to treatment [8,9,10,11,12,13]. The common challenges faced by surgeons and oncologists included the prioritization of patients according to their risk and urgency, the adaptation of surgical techniques and protocols to minimize hospital stays and complications, the coordination of multidisciplinary teams and resources, and the implementation of telemedicine and remote follow-up. The COVID-19 pandemic has also raised ethical dilemmas regarding the balance between providing optimal care for BC patients and protecting them and healthcare workers from infection. Moreover, the pandemic has disrupted ongoing clinical trials and research activities in BC surgery, as well as educational and training opportunities for surgeons [14,15,16,17]. According to a multicenter study, the COVID-19 pandemic forced surgical oncologists to change their daily practice and adopt alternative de-escalation strategies as the situation deteriorated. However, physicians have been instinctively reluctant to abandon standard criteria of care whenever possible [18]. An observational study conducted in an Italian academic hospital examined the impact of the pandemic on BC patients scheduled for surgery. The study found that 71% of patients reported increased stress and anxiety, 23% had difficulty contacting their doctor, and 18% had surgery delays [19].

Another Italian multicenter study evaluated the impact of the pandemic on breast cancer patients undergoing neoadjuvant therapy (NACHT). The study showed a reduction in the number of patients undergoing NACHT, with no changes in terms of indications, clinical presentation, and tumor response [20].

By this scenario, in a previous report about the experience of our institution, we analyzed the trend of oncological surgical resections during the most critical and dramatic period of the pandemic (9 March 2020 to 8 May 2020) by making a comparison with the same period of the previous 3 years. Not surprisingly, BC surgery showed the largest drop in surgical interventions (109 vs. 160; −31.9%) compared with other tumor types [21]. Given these premises, the purpose of this study is to evaluate the impact of COVID-19 on the surgical management of BC in our referral center by comparing the two pandemic years (2020–2021) with the two years before the SARS-CoV-2 outbreak.

## 2. Materials and Methods

### 2.1. Study Design and Study Population

Our main objective was to investigate the impact of the COVID-19 outbreak on the time interval between BC diagnosis and surgery and the pathological characteristics of the tumors. Moreover, we wanted to assess whether there were any differences in the type of surgical procedures (more or less conservative) or the indication for NACHT during the pandemic. In our single-institution retrospective study, we analyzed all cases of operable BC diagnosed at the Pathology Unit in “Città della Salute e della Scienza” in Turin, the largest academic hospital in Piedmont region, and the fourth largest nationwide (Italy), making a comparison between the 2018–2019 pre-pandemic period and the 2020–2021 pandemic period. The following clinical and pathological data were retrieved from medical charts: age at diagnosis, if BC was diagnosed through screening or not, date of histological diagnosis, date of surgery, clinical stage at diagnosis, type of breast surgery (conservative vs. mastectomy), axillary surgery (sentinel lymph node biopsy or axillary dissection), tumor invasiveness (in situ vs. invasive tumor), tumor grade (G1–2 vs. G3), tumor size (mm), histotype, estrogen receptors (ER), progesterone receptors (PR), HER2 and Ki67 expression, pathological T and N stage, NACHT.

Regarding tumor histotype, we divided the BCs of our cohort into non-special type (NST) and lobular cancers according to the WHO Classification of Tumors, 5th Edition [22]. All BCs that were not classifiable as NST or lobular were included in a third group. Tumor size was dichotomized by 15 mm, as suggested by previous studies [23,24,25].

The cut-off for ER and PR positivity was determined at <1%, according to the Consensus of St. Gallen 2011 [26]. HER2 was evaluated as recommended by the American Society of Clinical Oncology/College of American Pathologists [27]. Ki67 proliferation index was assessed on surgical specimens evaluating a minimum of 1000 cells. Luminal subtypes were defined according to St. Gallen’s proposal using a Ki67 cut-off value of 20%, in line with previously published studies [25,28,29]. BC was divided into five molecular subtypes based on ER, PR, Ki67 expression, and HER2 expression/gene amplification: luminal A-like, luminal B-like (HER2 negative), luminal B (HER2 enriched), HER2 positive (non-luminal), and triple negative. We administered NACHT to patients with advanced-stage BC or specific subtypes (triple negative, HER2 positive tumors). We selected cytotoxic chemotherapy and targeted biological therapies based on the tumor characteristics and the stage at diagnosis. Clinical and pathological staging was performed based on the TNM classification of malignant tumors, 8th edition [30]. We estimated the time between radiological diagnosis and surgery as the difference between the date of the mammography and the date of surgical intervention. All the cases were pseudonymously recorded into a dedicated database.

### 2.2. Statistical Analysis

Statistical analyses were performed using IBM^®^ SPSS^®^ v.23 (SPSS Inc., Chicago, IL, USA) software. Data were analyzed descriptively and represented as counts and percentages. All qualitative variables were analyzed through the Chi-square test. Bonferroni correction for multiple comparisons was used. Quantitative variables were reported as a median, range, and standard deviation (SD). Kolmogorov–Smirnov test was used to test data normality, and then the Mann–Whitney *U*-test was used for data comparison. Analyses were conducted with a 95% confidence interval (CI), and a *p*-value of 0.05 was considered statistically significant. All statistical tests were two-tailed.

### 2.3. Ethical Approval

This study was approved by the Research Ethics Committee for Human Biospecimen Utilization (Department of Medical Sciences—ChBU) of the University of Turin (n° 9/2019). This study was conducted by The Code of Ethics of the World Medical Association (Declaration of Helsinki).

## 3. Results

### 3.1. Clinicopathological Features of the Entire Study Population

In this study, we collected and analyzed data from 1331 BC patients who received surgical treatment at our institution from January 2018 to December 2021. The patients’ mean age at diagnosis was 62 years (SD ± 13, range 25–91), and 18% of them were diagnosed through screening. The mean intervals between histological diagnosis performed by tumor biopsy and surgical treatment were 69 days (SD ± 25, range 11–136) for BC in situ and 67 days (SD ± 26, range 11–155) for invasive tumors. Most of the patients were at clinical stage 0–I–II at the time of diagnosis and underwent conservative surgery (67%), while sentinel lymph node biopsy was performed for lymph node staging in 84% of cases. Most of the tumors were invasive (86%) and had a diameter of ≥15 mm (52%) with grades 2–3 (83%). As expected, most of the tumors were NST (66%) with a luminal A molecular profile (54%). NACHT was administered to 20% of the patients. Among patients undergoing surgery without NACHT, the majority were staged as pT1 (73%) tumors and/or pN0 (74%); similarly, patients who underwent NACHT predominantly showed a ypT1 (45%) and/or a ypN0 (58%) stage after surgery. All demographic, clinical, and pathological features of the whole cohort are summarized in Table 1.

### 3.2. Comparison of Clinicopathological Features between the Pre-Pandemic and Pandemic Periods

Overall, 726 patients with BC were surgically treated in the pre-pandemic years vs. 605 patients in the pandemic period (−121, 9%). The median age of patients with BC was slightly higher in the pandemic period (61 years SD ± 13, range 26–89 vs. 64 years SD ± 14, range 25–91, *p*-value = 0.03). We did not find any differences between patients who were diagnosed with BC within the screening program and those who were diagnosed outside the screening program. We also did not find any significant differences in the median time (days) from the histological diagnosis to the surgical resection for both in situ and invasive BC. The type of surgical treatment performed did not vary, maintaining a constant ratio between conservative surgery, which was the most common one (67% in both groups), and mastectomy. Therefore, the surgical management and the alternative surgical options were similar for the patients in the pre-COVID and COVID two-year periods. However, we observed a significant decrease in the number of patients who underwent axillary dissection during the pandemic (21% vs. 8%, *p* = < 0.001). Even though no difference was detected in terms of the median size of invasive BC, the number of patients presenting a higher pT on the surgical specimen (pT2 and pT3) in the two-year COVID period was significantly superior to the one of the pre-pandemic period (6% vs. 3%, *p* = 0.03), albeit exclusively in the subgroup of invasive tumors that had not received NACHT.

The most frequent histotype was NST, while the other special histotypes constituted 34% of cases in 2018–2019 and 30% in 2020–2021, with a prevalence of lobular BC.

Regarding molecular subtypes, luminal A was the most frequent one in both groups, whereas, in the pandemic period, a statistically significant decrease in luminal B was observed (22% 2020–2021 vs. 30% 2018–2019, *p* = 0.015). Concerning grading, a decrease in grade 1 and an increase in higher grade (2–3) tumors was observed in the years 2020–2021 (20 % grade 1 in 2020–2021 vs. 27% in 2018–2019; 80% grade 2–3 in 2020–2021 vs. 73% in 2018–2019).

An additional indicator of interest was NACHT: data were similar (90% of patients treated with NACHT in 2020–2021 vs. 89% in 2018–2019) without observing differences in pathological staging in this subgroup of patients. All the characteristics of the BC according to the pandemic and pre-pandemic period are summarized in Table 2.

## 4. Discussion

The COVID-19 pandemic has disrupted the whole spectrum of cancer care, leading to diagnostic and therapeutic delays and hampering clinical trials. According to a global collaborative study, the pandemic has caused a widespread detrimental impact on cancer care, with varying magnitude among centers worldwide. The most common disruptions reported were postponement or cancellation of elective surgery, chemotherapy, radiotherapy, immunotherapy, screening programs, follow-up visits, and supportive care. The reasons for these disruptions included overwhelmed health systems, lack of personal protective equipment, staff shortages, restricted access to medications, and infection control measures. The disruption of cancer care can lead to worse outcomes for cancer patients, such as disease progression, increased morbidity and mortality, and reduced quality of life [31].

In a large cross-sectional study that looked at the number of new cancers diagnosed before and after the COVID-19 pandemic in the United States, a reduction in newly diagnosed cancers was observed for six common types of tumors (breast, colorectal, lung, pancreatic, gastric, and esophageal) [31]. In particular, BC is the tumor type with the greatest decline in newly diagnosed cases (51.8%, from 2208 to 1064 new cases; *p* < 0.001) [32]. The causes of this serious collapse of new BC diagnoses may include the lockdown, social distancing, fear of accessing hospital facilities, and the reduction in medical staff dedicated to BC management. In addition, there has been a dramatic reduction in access to preventive screening, an issue that has also been observed in our country [33,34].

In our breast unit, a 9% reduction in BC patients who underwent surgery was observed. This reduction was lower than the preliminary estimate of 31.9% reported by our group [21] and lower than in another recent Italian multicenter study [35]; however, in the present study, we considered not only 2020 but also 2021, so the impact of COVID-19 was probably greater in the first months of lockdown compared with the longer time interval now considered. The authors of the previous study also reported that the decline in BC diagnosis had reached its highest value (26%) after the flattening of the first epidemic curve and the resumption of cancer screenings (June 2020) [35]. It is interesting to note that, despite a reduction in BC screening program activity, in our study, the proportion of cases diagnosed between the pandemic and the pre-pandemic period remained constant.

The median time from diagnosis to surgery was always less than 90 days regardless of the period considered and the invasiveness of the tumor, which represents, according to the literature, the biological cut-off potentially leading to a poorer prognosis [36,37]. In some patients with invasive BC, the time between diagnosis and surgery was over 100 days, but in most of these cases, the delay was due to patient characteristics and not because of pandemic-related events. For example, in elderly patients with large tumors and important comorbidities, it was preferred to first administer hormone therapy for an average of three to four months and then re-evaluate the surgery feasibility. In contrast to other studies [34,35], we did not observe an increase in cases of late-stage disease at presentation compared to the pre-pandemic period. However, this finding could be due to the study focus (i.e., surgically treated tumors only) and/or to the longer period analyzed, which could have balanced the impact of the initial critical months of the pandemic. Regarding the type of surgery, we found no differences between conservative surgery and mastectomy rates; however, we did observe a significant reduction in axillary dissections during the pandemic period. A similar result was also observed in the study proposed by the Italian Senonetwork [38], which observed a greater number of cases staged with sentinel node biopsy compared to axillary dissection. The authors of this study suggest that the lower rate of axillary dissections could be due to an increase in cN0 cases selected for upfront surgery (actually, in our study, we have an increase in pN0, albeit not significant) and probably reflects the paradigm shift due to the progressive acceptance of the Z0011 trial findings on the possibility of omitting axillary dissection in selected low-risk tumors [39].

Furthermore, axillary dissection is associated with higher morbidity and complications, such as lymphedema, pain, infection, nerve injury, and reduced shoulder mobility, and may not provide additional prognostic or therapeutic benefit for patients with limited nodal involvement, especially those who receive systemic therapy and/or radiotherapy [40]; also, in this specific setting, it finally may have increased the risk of exposure to COVID-19.

Regarding the pathological features of the analyzed BC, in the pandemic period, we detected a significant increase in higher grade (2–3) tumors, pT stage 3–4 BC without previous NACHT, and a reduction in luminal B tumors. No significant differences in invasiveness, tumor diameter, histotypes, and pT and pN after NACHT were observed.

Overall, these data may suggest an increase in locally advanced tumors that have been surgically treated upfront, probably considering their biological characteristics not suitable for NACTH (e.g., locally advanced luminal A tumors). In addition, despite a non-significant difference, we observed a reduction in the number of NACHTs administered in the pandemic period (69 vs. 53, *p* = 0.606), a finding that may help explain the increase in pT4 tumors. A significant decrease in NACHT utilization has also been observed by other authors analyzing a shorter time interval [38]. These data are in contrast with some international guidelines which promoted the use of neoadjuvant treatments. However, there are also some challenges and limitations associated with NACHT during the pandemic, such as the lack of standardized criteria for patient selection, treatment duration, response evaluation, and surgical planning; the variability in the availability and accessibility of NACHT across different settings; the uncertainty about the long-term efficacy and safety of NACHT compared with immediate surgery; and the potential toxicity and immunosuppression caused by NACHT [41,42].

Another strategy to mitigate the possible negative impact of surgical delay is neoadjuvant endocrine therapy (NET). NET showed several advantages during the pandemic: first, NET can be administered orally at home, avoiding hospital visits and exposure to COVID-19. Second, NET has a favorable toxicity profile, with fewer side effects and lower immunosuppression than NACT, reducing the risk of complications and infections. Third, NET can achieve tumor shrinkage and downstaging in a significant proportion of patients, especially those with low-grade and low-proliferative tumors, facilitating breast-conserving surgery and reducing the need for axillary dissection. Fourth, NET can provide prognostic information based on the response to treatment, allowing for personalized therapy and de-escalation of adjuvant therapy.

Several national and international guidelines and consensus statements have recommended the use of NET for ER+ BC patients during the pandemic, especially for those with early-stage or operable locally advanced disease who were not candidates for NACHT or immediate surgery. The preferred drugs for NET are tamoxifen for premenopausal women and aromatase inhibitors for postmenopausal women [43,44,45,46].

Several studies have reported the impact of NET on BC surgery during the pandemic in different countries and regions [43,44,45,46]. The results showed that NET has been widely adopted as a strategy to defer surgery safely and effectively, with no major differences in surgical outcomes compared with the pre-pandemic period. However, there are also some challenges and limitations associated with NET, such as the lack of standardized criteria for patient selection, treatment duration, response evaluation, and surgical planning; the variability in the availability and accessibility of NET across different settings; the uncertainty about the long-term efficacy and safety of NET compared with immediate surgery; and the potential psychological distress and anxiety caused by delaying surgery [44,47]. In our Breast Unit, given that the times between diagnosis and surgery have been respected, this strategy has not been adopted.

This study has some strong points and limitations that should be considered. The main merits of this study are related to the fact that it examined a large group of consecutive BC patients who received treatment in a large referral center deployed in a real-world setting. On the other hand, the major limitations are associated with the unvoidable retrospective design of this study, although all BC cases were managed by dedicated and experienced physicians who participated in regular multidisciplinary tumor boards despite the ongoing pandemic. Other researchers have reported a 13% decrease in the number of multidisciplinary BC cases that were discussed after the pandemic (60% vs. 73%, *p* < 0.01) [35]; however, our breast unit quickly adapted the discussion of the BC cases to an online format to preserve the multidisciplinary consultations and provide the best possible standard of care [48].

## 5. Conclusions

The SARS-CoV-2 pandemic had a devastating impact on healthcare services in several countries around the world, and the management of some diseases, such as BC, has particularly suffered from the reorganization put in place to address the pandemic. Many authors have analyzed the impact of SARS-CoV-2 in the pandemic’s most intense period, confirming a reduction in diagnoses and changes in the management of BC [8,9,10,11,12,13,35].

However, our study reports that by comprehensively analyzing the most critical two years of the pandemic (2020–2021), the difference in the number of surgically treated cases had been limited (9%), and the management of tumors has not undergone major differences (except for fewer axillary dissections and a relatively small increase in pT4 cases not subjected to NACT), suggesting a prompt resumption of a surgical activity similar to the pre-pandemic period. To date, our study is one of the few that has evaluated the impact of the pandemic on BC surgery over a long period of time, covering both the first and second wave of COVID-19 infections. Most of the previous studies have focused on a shorter time frame, mainly including the first wave, when the lockdown measures were stricter, and the screening programs were suspended or postponed. Therefore, our study provides a more comprehensive picture of how BC surgery has adapted to the changing scenario and how it has recovered after the initial disruption.

One of the main challenges faced by BC surgeons during the pandemic was to prioritize patients according to their risk and urgency while minimizing hospital stays and complications. To achieve this goal, several strategies were implemented, such as using NACHT to delay surgery for patients with high-risk tumors or operable locally advanced disease, performing breast-conserving surgery instead of mastectomy when feasible, avoiding axillary dissection for patients with clinically negative nodes or low-volume disease, and using telemedicine and remote follow-up for postoperative care.

These strategies were consistent with the recommendations issued by several consensus groups [41,49,50]. However, there was also some variability in how these recommendations were applied in different settings and regions, depending on the local resources and policies. Therefore, it is important to gather data and compare the outcomes of BC surgery during and after the pandemic across different centers and countries, as well as to evaluate the long-term effects of these changes on patients’ survival and quality of life.

We hope that our findings will contribute to improving BC surgery practice in the face of future pandemics or similar emergencies.

## Figures and Tables

**Table 1:** Patient’s characteristics of the entire period (2018–2022).

Variable	All Patients (N = 1331)
Median age (years) SD (range)	62 ± 13 (25–91)
Screening	
Yes	237 (18%)
No	1094 (82%)
Median time (days) between radiological diagnosis and surgery (in situ tumors) SD (range)	69 ± 25 (11–133)
Median time (days) between radiological diagnosis and surgery * (excluding NACHT) SD (range)	67 ± 26 (11–155)
Clinical stage at diagnosis	
Stage 0–I–II	1266 (95%)
Stage III	65 (5%)
Breast surgery	
Conservative	895 (67%)
Mastectomy	436 (35%)
Axillary dissection *	
No	970 (84%)
Yes	178 (16%)
Invasiveness	
In situ	183 (14%)
Invasive	1148 (86%)
Diameter (mm) *	
<15	556 (48%)
≥15	592 (52%)
Grade *	
1	270 (27%)
2–3	878 (73%)
Histotype *	
NST	779 (66%)
Lobular	175 (16%)
Others	194 (18%)
Molecular profile *	
Luminal A	635 (54%)
Luminal B	305 (30%)
HER2+ HR+	69 (5%)
HER2+ HR−	28 (2%)
Triple-negative	111 (9%)
NACHT *	
No	1026 (77%)
Yes	122 (33%)
Pathological T stage (excluding NACHT) *	
1	750 (73%)
2	229 (22%)
3–4	47 (5%)
Pathological N stage (excluding NACHT) *	
0	408 (74%)
1	107 (19%)
2–3	40 (7%)
Pathological T stage (considering only NACHT) *	
0	35 (29%)
1	55 (45%)
2	26 (21%)
3–4	6 (5%)
Pathological N stage (considering only NACHT) *	
0	71 (58%)
1	31 (26%)
2	12 (10%)
3	8 (6%)

* For this subgroup, only invasive tumors were considered. HR: hormone receptor; NACHT: neoadjuvant chemotherapy; SD: standard deviation.

**Table 2:** Patients’ characteristics according to the pandemic and pre-pandemic periods.

Variable	Years 2018–2019 (N = 726)	Years 2020–2021 (N = 605)	*p*-Value
Median age (years) SD (range)	61 ± 13 (26–89)	64 ± 14 (25–91)	**0.002**
Screening			
Yes	133 (18%)	104 (17%)	0.529
No	593 (82%)	501 (83%)	
Median time (days) between radiological diagnosis and surgery (in situ tumors) SD (range)	67 ± 25 (17–133)	72 ± 25 (11–136)	0.238
Median time (days) between radiological diagnosis and surgery * (excluding NACHT) SD (range)	67 ± 27 (12–154)	66 (11–155)	0.66
Clinical stage at diagnosis			
Stage 0–I–II	688 (95%)	578 (96%)	0.56
Stage III	38 (5%)	27 (4%)	
Breast surgery			
Conservative	490 (67%)	405 (67%)	0.831
Mastectomy	236 (33%)	200 (33%)	
Axillary dissection			
No	488 (79%)	482 (92%)	**<0.001**
Yes	136 (21%)	42 (8%)	
Invasiveness			
In situ	102 (14%)	81 (13%)	0.727
Invasive	624 (86%)	524 (87%)	
Diameter (mm) *			
<15	299 (48%)	257 (49%)	0.703
≥15	325 (52%)	267 (51%)	
Grade *			
1	166 (27%)	104 (20%)	**0.007**
2–3	458 (73%)	420 (80%)	
Histotype *			
NST	412 (66%)	367 (70%)	0.266
Lobular	97 (16%)	78 (15%)	
Others	115 (18%)	79 (15%)	
Molecular profile *			
Luminal A	337 (54%) _a_	298 (57%) _a_	**0.015**
Luminal B	**188 (30%) _a_**	**117 (22%) _b_**	
HER2+ HR+	31 (5%) _a_	38 (7%) _a_	
HER2+ HR−	11 (2%) _a_	17 (3%) _a_	
Triple-negative	57 (9%) _a_	54 (10%) _a_	
NACHT *			
No	555 (89%)	471 (90%)	0.606
Yes	69 (11%)	53 (10%)	
Pathological T stage (excluding NACHT) *			
1	407 (73%) _a_	343 (73%) _a_	**0.03**
2	131 (24%) _a_	98 (21%) _a_	
3–4	**17 (3%) _a_**	**30 (6%) _b_**	
Pathological N stage (excluding NACHT) *			
0	408 (74%)	363 (77%)	0.411
1	107 (19%)	80 (17%)	
2–3	40 (7%)	28 (6%)	
Pathological T stage (considering only NACHT) *			
0	16 (23%)	19 (36%)	0.422
1	32 (46%)	23 (43%)	
2	17 (25%)	9 (17%)	
3–4	4 (6%)	2 (4%)	
Pathological N stage (considering only NACHT) *			
0	36 (52%)	35 (66%)	0.410
1	19 (27%)	12 (23%)	
2	8 (12%)	4 (7%)	
3	6 (9%)	2 (4%)	

* For this subgroup, only invasive tumors were considered. HR: hormone receptor; NACHT: neoadjuvant chemotherapy; SD: standard deviation. Significant *p*-values in bold. Subscripted letters indicate differences between subgroups estimated with Bonferroni’s correction.

## Data Availability

Data are encrypted and are available on reasonable request from the authors.
